# A circRNA-miRNA-mRNA network plays a role in the protective effect of diosgenin on alveolar bone loss in ovariectomized rats

**DOI:** 10.1186/s12906-020-03009-z

**Published:** 2020-07-14

**Authors:** Zhiguo Zhang, Lifeng Yue, Yuhan Wang, Yanhua Jiang, Lihua Xiang, Yin Cheng, Dahong Ju, Yanjing Chen

**Affiliations:** 1 Institute of Basic Theory, China Academy of Chinese Medical Sciences, Beijing, 100700 China; 2 Dongzhimen Hospital, Beijing University of Chinese Medicine, Beijing, 100700 China; 3 Experimental Research Center, China Academy of Chinese Medical Sciences, Beijing, 100700 China

**Keywords:** Diosgenin, Alveolar bone, Osteoprotective effect, CircRNAs, Coexpression network

## Abstract

**Background:**

The present study aimed to assess the perturbation in circular RNA (circRNA)/mRNA expression profiles and a circRNA-miRNA-mRNA coexpression network involved in the potential protective effect of diosgenin (DIO) on alveolar bone loss in rats subjected to ovariectomy (OVX).

**Methods:**

The Wistar rats (female) manipulated with sham operation were classified as the SHAM group and the grouping of OVX rats administered with DIO, estradiol valerate or vehicle for 12 weeks was DIO group, EV group and OVX group respectively. Following treatments, the plasmatic levels of osteocalcin and tumor necrosis factor-alpha and the microstructure of alveolar bone were assayed. Based on microarray analyses, we identified differentially expressed (DE) circRNAs and mRNAs in alveolar bone of rats in both OVX and DIO group. The DE circRNAs and DE mRNAs involved in the bone metabolism pathway validated by RT-qPCR were considered key circRNAs/mRNAs. On the basis of these key circRNAs/mRNAs, we predicted the overlapping relative miRNAs of key circRNAs/mRNAs, and a circRNA-miRNA-mRNA network was built.

**Results:**

DIO showed an anti-osteopenic effect on the rat alveolar bone loss induced by OVX. In total, we found 10 DE circRNAs (6 downregulated and 4 upregulated) and 614 DE mRNAs (314 downregulated and 300 upregulated) in samples of the DIO group compared with those of the OVX group. However, only one circRNA (rno_circRNA_016717) and seven mRNAs (*Sfrp1*, *Csf1*, *Il1rl1*, *Nfatc4*, *Tnfrsf1a*, *Pik3c2g*, and *Wnt9b*) were validated by qRT-PCR and therefore considered key circRNA/mRNAs. According to these key circRNA/mRNAs and overlapping predicted miRNAs, a coexpression network was constructed. After network analysis, one circRNA-miRNA-mRNA axis (circRNA_016717/miR-501-5p/*Sfrp1*) was identified.

**Conclusion:**

The mechanism of DIO inhibiting alveolar bone loss after OVX is possibly relevant to the simultaneous inhibition of osteogenesis and osteoclastogenesis by mediating the expression of important molecules in the Wnt, PI3K, RANK/RANKL or osteoclastogenic cytokine pathways. The circRNA_016717/miR-501-5p/*Sfrp1* axis may play important roles in these processes.

## Background

Tooth and periodontal diseases associated with alveolar bone loss are high risk complications for and frequently occur in postmenopausal women suffering from osteoporosis because of estrogen deficiency [1, 2]. Unlike other bones, the biological metabolic cycle of an alveolar bone is much shorter [3], but this bone is more tolerant to estrogen deficiency than appendicular long bones [4]. Some studies have suggested that estrogen can protect against alveolar bone loss not only in humans [5] but also in rodents [6].

Many active constituents in plants, especially steroid saponins, are thought to have estrogen-like effects and are called phytoestrogens [7]. Diosgenin (DIO), a phytosteroid sapogenin, is commonly categorized as a phytoestrogen [8–10]. The results regarding the estrogen-like effect of DIO have been controversial. A study suggested that DIO (20–200 mg/kg) had no effect on the epithelium height, uterine weight, volume density of endometrium, endometrial/vaginal epithelia or endometrial glands in rats [11]. Nonetheless, other researchers have found that DIO had adverse effects. For example, in ovariectomized (OVX) mice, DIO may stimulate the growth of mammary glands [12]. Regarding bone metabolism, our previous studies showed that decreasing the ratio of receptor activator of nuclear factor-kappa B ligand (RANKL)/osteoprotegerin (OPG) of the tibia [13] or regulating the expression of long noncoding RNAs in alveolar bone [10] was the reason for the anti-bone loss effect of DIO in OVX rats.

Compared with linear RNA (e.g., mRNA, miRNA, lncRNA) characterized by 3′ tale and 5′ cap structures, circular RNA (circRNA) features a covalently closed continuous loop generated by a unique splicing strategy [14]. CircRNAs are thought to participate in various diseases because of their special “miRNA sponge” function, whereby they bind miRNAs to efficiently suppress miRNA transcription, and this inhibitory effect on miRNAs can further regulate the expression of downstream mRNAs [15]. In bone metabolism, circRNAs play an important role in modulating mRNA expression via the circRNA-miRNA-mRNA axis not only in a murine model [16] but also in humans [17]. Thus, we raise the question of whether the regulatory effect of DIO on bone metabolism was mediated via circRNA-miRNA-mRNA interactions. To answer this question, we carried out the present study to explore the action of DIO on gene profiles or the circRNA-miRNA-mRNA network in the alveolar bone of OVX rats.

## Methods

### Experimental animals and treatments

Six-month-old female rats undergoing ovariectomy have been commonly used as a model to study osteoporosis in postmenopausal women [18, 19]. This study included 48 female Wistar rats (six months old). We obtained the rats (average weight 300 ± 20.0 g) from the National Institutes for Food and Drug Control of China. All rats were kept under a regular 12 h/12 h light/dark cycle and at constant room temperature (22 ± 1 °C). The rats underwent sham operation (SHAM, *n* = 12) or bilateral OVX (*n* = 36). The ventral approach was used in bilateral ovariectomy [20]. Briefly, at first, the anesthetized rats were fixed ventrally and the abdominal skin in the mid-lower back was preparated and disinfected. A 0.5 cm incision closed the spine under the costal margin was made and skin was separated. Secondly, dorsal muscle was cut and then ovary was exposed adequately. Thirdly, the fat tissue coating the ovary was separated by blunt dissection and the blood vessels and oviduct were ligated. Fourthly, ovariectomy was completed and the preserved tissue or organs were put back into the abdominal cavity, followed by suturing incision. Ovariectomy on the other side was in the same way. The SHAM group rats underwent same operation except that the ovaries were preserved.

The model rats subjected to OVX were classified into 3 categories randomly, namely, OVX group (*n* = 12), estradiol valerate (EV) group (*n* = 12) and DIO group (*n* = 12). According to studies published previously [21–23], EV group rats were administered oral gavage of EV (0.1 mg/kg body weight, Bayer Health Care, Guangzhou, China) every day, while DIO group rats were treated by oral gavage with DIO (100 mg/kg body weight, purity≥93%, Sigma-Aldrich, Saint Louis, MO, USA) every day. For the SHAM or OVX group, rats were administered distilled water (of equal volume) by oral gavage. A standard chow was used to feed all rats during our present experiment. The treatments started one week after the operation and lasted 12 weeks. No rats died during the whole treatment period.

### Specimen preparation

At the end of treatment, all experimental rats were anesthetized via intraperitoneal injection by ketamine (80 mg/kg) and xylazine (12 mg/kg) and were subsequently exsanguinated for sacrifice. Under anesthesia, we punctured the abdominal aortae to collect blood (8–10 mL) which was subsequently transferred into tubes added with heparin. The rats subjected to haemospasia were palpated for 5 min to ensure asystole and were considered as dead after confirming asystole, respiration cease and corectasis. Next, the plasma was separated from blood by centrifugation (12 min, 2500 g, 4 °C) and stored (− 80 °C) for the following experiments. To observe and determine the alveolar bone microstructure of the rats, the right mandibles were first excised and kept under the temperature of − 20 °C and then scanned using microcomputed tomography (micro-CT). Thereafter, the right mandibles also served as the specimens for histological examination. For microarray assays and reverse transcription-quantitative polymerase chain reaction (RT-qPCR), we used osseous tissue between the first molar and incisor in left mandibles.

### Enzyme-linked immunosorbent assays (ELISA)

We used ELISA to determine plasma concentrations of osteocalcin (OCN) and tumor necrosis factor-alpha (TNF-alpha). A rat OCN ELISA kit (Novus Biologicals, Littleton, CO, USA) and a rat TNF-alpha ELISA kit (Abcam, Cambridge, MA, USA) were used to determine the rat plasma levels of TNF-αand OCN from all groups. The absorption value at 450 nm was detected by a BioTek ELISA reader (BioTek Instruments., Winooski, VT, USA).

### Micro-CT scanning

The micro-CT scanning method has been previously described [24]. The right mandibles of the rats were scanned by using a high-resolution micro-CT system (Skyscan 1174, Bruker Corporation, Ettlingen, Germany) without any sample processing. The resolution of the scan was 9.8 μm, and analuminum filter (0.5 mm) was used to remove image noise. A global threshold (upper gray threshold: 255, lower gray threshold: 55) was used for the quantity of the parameters about trabecular bone.

The image capture conditions were 800 μA and 50 keV. A cube (0.5 mm × 0.5 mm × 0.5 mm) termed as “volume of interest” (VOI) was reconstructed starting from 0.5 mm beneath the base of crown in the first molar. The trabecular bone morphological characteristics within the VOI were measured applying the Skyscan software package. Furthermore, a 3-D analysis was performed to determine eight key parameters, namely, the trabecular bone volume fraction (BV/TV), the bone surface (BS), the trabecular separation (Tb.Sp), the trabecular number (Tb.N), the trabecular thickness (Tb.Th), the trabecular pattern factor (Tb.Pf), the degree of anisotropy (DA) and the structural model index (SMI) of the identical VOL [25].

### Observations of histology

The prepared solution containing formalin (10%) was used to fix the right mandibles. Next, the mandibles were decalcified with ethylenediaminetetraacetic acid (EDTA, 14%) and embedded in paraffin. After a regular microtome cutting process, the glass slides were used to affix sections, which then were stained with hematoxylin and eosin.

### CircRNA and mRNA microarray analysis

Twelve samples of alveolar bones in the DIO group (*n* = 6) and OVX group (*n* = 6) were chosen for microarray studies randomly. The microarray assay was completed by KangChen Biotech Inc. (Shanghai, China).

For our circRNA study, we used an Arraystar Rat circRNA Array (Arraystar, Rockville, MD, USA). We used RNase R (Epicentre, Madison, WI, USA) to digested total RNA, and further remove linear RNA and concentrate circular RNA. Subsequently, we amplified and transcribed the concentrated circRNA into fluorescent RNA. This step was performed using an Arraystar Super RNA Labeling Kit (Arraystar, Rockville, MD, USA), by a random priming method. Thereafter, the labeled cRNA was subjected to hybridization, washing and scanning on Arraystar mouse circRNA Array v1.0 using DNA Microarray Scanner (Agilent Technologies, Santa Clara, CA, USA).

For mRNAs, we used a rat 4 × 44 K Gene Expression Array (Agilent Technology, Santa Clara, CA, USA). In line with the protocol, samples were labeled and arrays were hybridized. Then, the obtained array images were evaluated by the Agilent Feature Extraction software (version 11.0.1.1). The data was normalized and further processed using an R software package.

CircRNAs/mRNAs with fold change≥1.5 and *P*-value< 0.05 were regarded as differentially expressed (DE) circRNAs/mRNAs.

### Analysis of ingenuity pathway analysis (IPA)

All DE mRNAs obtained from our array analyses were input into the IPA system, a system that predicts canonical pathways, global functions and biological networks of a particular gene dataset and is referred to as the Ingenuity Pathways Knowledge Base (IPKB). In IPKB, “Role of Osteoblasts, Osteoclasts and Chondrocytes in Rheumatoid Arthritis (ROOCRA)” pathway is a exclusive pathway because this pathway includes nearly all known pivotal molecules and signaling pathways that are closely related to the development, differentiation, degradation, mineralization and apoptosis of osteoblasts and osteoclasts. As this pathway is essential in bone metabolism, the DE mRNAs that belong to the ROOCRA pathway were therefore regarded as key DE mRNAs.

### Building of a circRNA-miRNA-mRNA coexpression network

Potential target miRNAs of circRNAs were predicted by miRanda (v3.3a) [26] and TargetScan (version 7.2) [27]. Software developed by Arraystar was applied to predict the interactive relationship between circRNAs and miRNAs, while the predicted interactions of mRNAs and miRNAs were obtained using miRanda (version 3.3a). After Pearson correlation analyses of the predicted mRNA-miRNA and circRNA-miRNA coexpression networks, the circRNA-miRNA-mRNA coexpression network were constructed. Cytoscape (version 2.8.2) [28] was applied to visualize the whole circRNA-miRNA-mRNA network.

### Validations of DE circRNAs, key DE mRNAs and predicted miRNAs using RT-qPCR

qRT-PCR assays used for detecting the expression levels of the predicted miRNAs were all performed in an ABI 7500 system (Applied Biosystems, Foster City, CA, USA) using SYBR RT-PCR kits (Takara, Dalian, Liaoning, China). We used the method of 2^-ΔΔCt^ cycle threshold, and the expression of *U6* served as the internal normalization control. For circRNAs/mRNAs expression data, the internal control was *Gapdh* expression.

### Statistical analysis

The mean ± standard deviation was used to express all values. We utilized SPSS 19.0 (SPSS Inc., Chicago, IL, USA) for analyzing data statistically. The difference of the assessed parameters between rats from two groups or four groups was tested using the the *t*-test or the one-way analysis of variance (ANOVA) and subsequently the least significant difference (LSD) test, respectively. Kolmogorov-Smirnov statistics was used to test the normality of data from all groups. Statistical significance was set at *p* < 0.05.

## Results

### Regulation on plasmic OCN and TNF-alpha by DIO

**Fig. 1:**
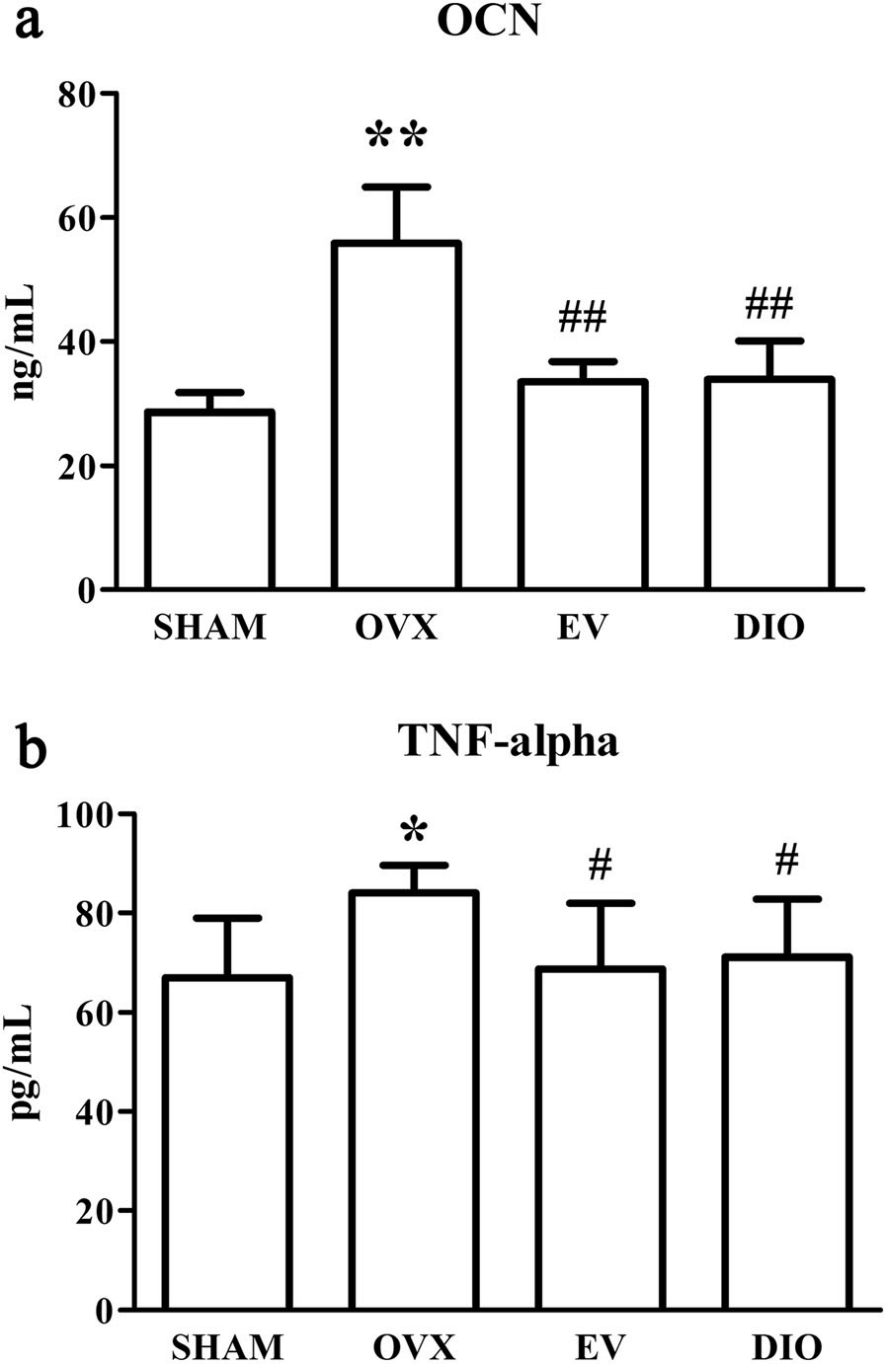
Effect of different treatments on OCN and TNF-alpha levels in plasma. (A) OCN, (B) TNF-alpha. ^#^*p* < 0.05 vs. the OVX group; *p < 0.05 vs. the SHAM group

Figure 1A and B showed the plasmic concentrations of OCN and TNF-alpha in the rats that were subjected to 12-week long treatment. After the treatment, the OCN and TNF-alpha contents of rats in the OVX group were remarkably superior to those of rats in the SHAM group (*p* < 0.01). Contrastively, the DIO and EV group rats exhibited significantly lower plasmic OCN and TNF-alpha levels than the OVX group rats (*p* < 0.05).

### Effects of DIO on the microstructure of trabecular bones

**Fig. 2:**
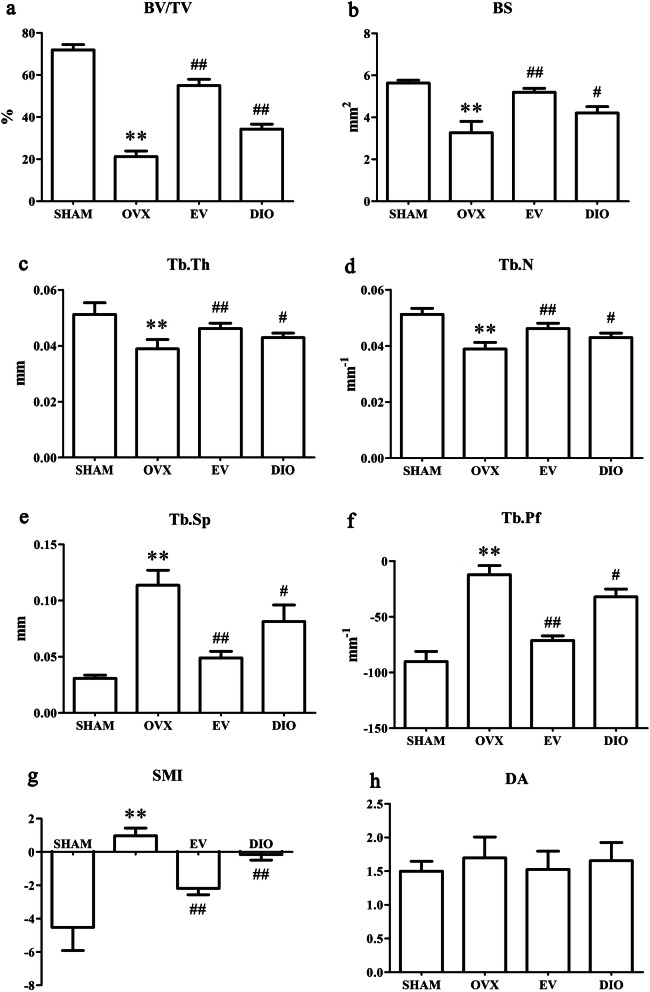
Effect of different treatments on trabecular bone microarchitecture. (A) BV/TV, (B) BS (C) Tb.Th, (D) Tb.N, (E) Tb.Sp, (F) Tb.Pf; (G) SMI, (H) DA. **p < 0.01 vs. the SHAM group; ^#^*p* < 0.05 vs. the OVX group; ^##^*p* < 0.01 vs. the OVX group

**Fig. 3:**
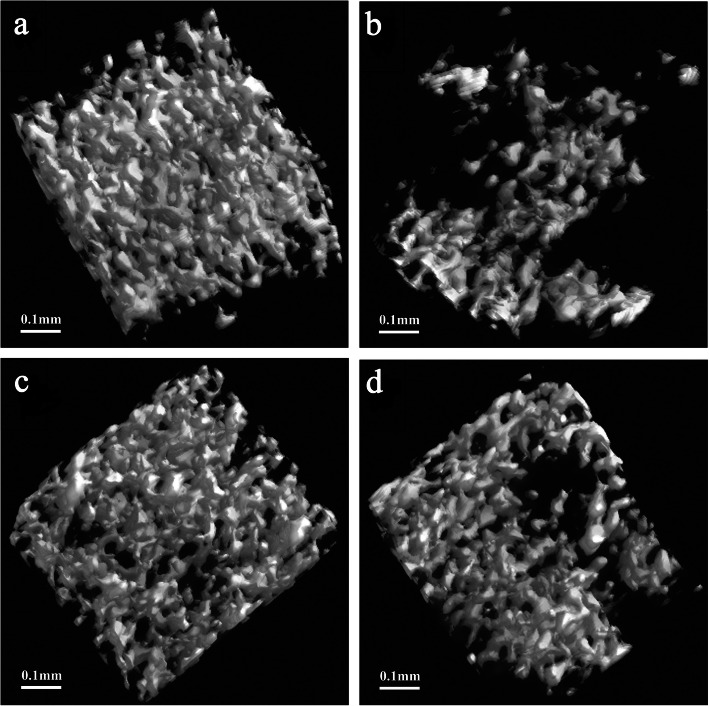
Representative three dimensional structures of alveolar bone beneath the first molar from each group. (A) SHAM group, (B) OVX group, (C) EV group and (D) DIO group

Our micro-CT results suggested that in the OVX group, morphological parameters of bone, such as BV/TV, BS, Tb.Th, and Tb.N were substantially reduced, yet Tb.Pf, Tb.Sp and SMI were raised in comparison with the SHAM group (Fig. 2A-H). As shown in Fig. 2, significant changes in the bone morphological parameters were found when the rats were treated with EV or DIO. Furthermore, trabecular impairment due to ovariectomy was lessened by DIO or EV treatment (Fig. 3A-D).

### Effect of DIO on histomorphological change of alveolar bone

**Fig. 4:**
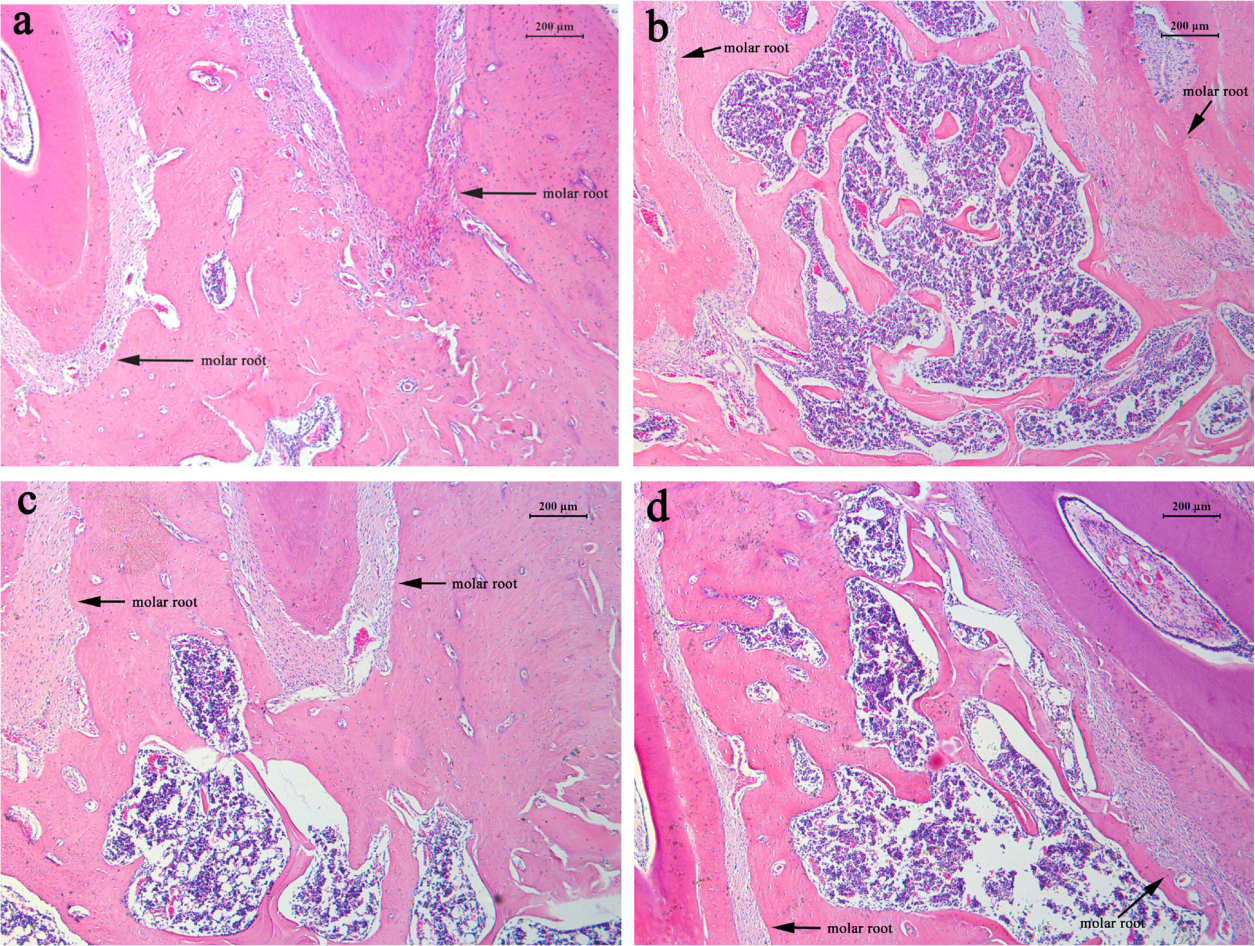
Observing alveolar bones beneath the first molar histomorphologically. (A) SHAM group, (B) OVX group, (C) EV group and (D) DIO group. The arrows indicate roots of the first molar. Original magnification, × 5, scale bar = 200 μm

To understand the effect of DIO from a histological perspective, we observed the alveolar bone beneath the first molar. Representative histomorphological images from four group rats are shown in Fig. 4. It is clear that the SHAM group rats had thicker trabeculae in alveolar bone and scant and smaller medullary cavity (Fig. 4A) in comparison to the OVX group rats. It appears that OVX decreases alveolar bone volume and increases the medullary cavity size (Fig. 4B). Furthermore, bone volume beneath the first molar was significantly enlarged after DIO or EV treatment (Fig. 4C and D), and EV appeared to have a stronger effect in this regard. With these data, our results from histological observations and micro-CT were consistent with each other.

### Regulation of the expression profiles of circRNA and mRNAs by DIO

Our microarray results revealed that there were, in total, 10 DE exonic circRNAs (Table 1) and 614 DE mRNAs (Table S4) in the alveolar bone between DIO and OVX group rats. Four and 6 circRNAs were up-and downregulated, respectively, while almost half of the mRNAs were increased and half were decreased (300 were upregulated and 314 downregulated).

**Table 1:** Differentially expressed exonic circRNAs of alveolar bone of rats in the DIO in comparison to those in the OVX group (*p*<0.05)

CircRNA Name	Fold Change
rno_circRNA_000034	2.493
rno_circRNA_016717	1.729
rno_circRNA_005275	1.537
rno_circRNA_007431	1.536
rno_circRNA_003113	−1.514
rno_circRNA_014839	−1.605
rno_circRNA_002656	−1.640
rno_circRNA_002387	−1.698
rno_circRNA_002043	−1.735
rno_circRNA_001963	−1.858

The pattern of presenting values is average value ± standard deviation (*n* = 6 for each group).

### Regulation of the bone metabolic pathway by DIO

**Fig. 5:**
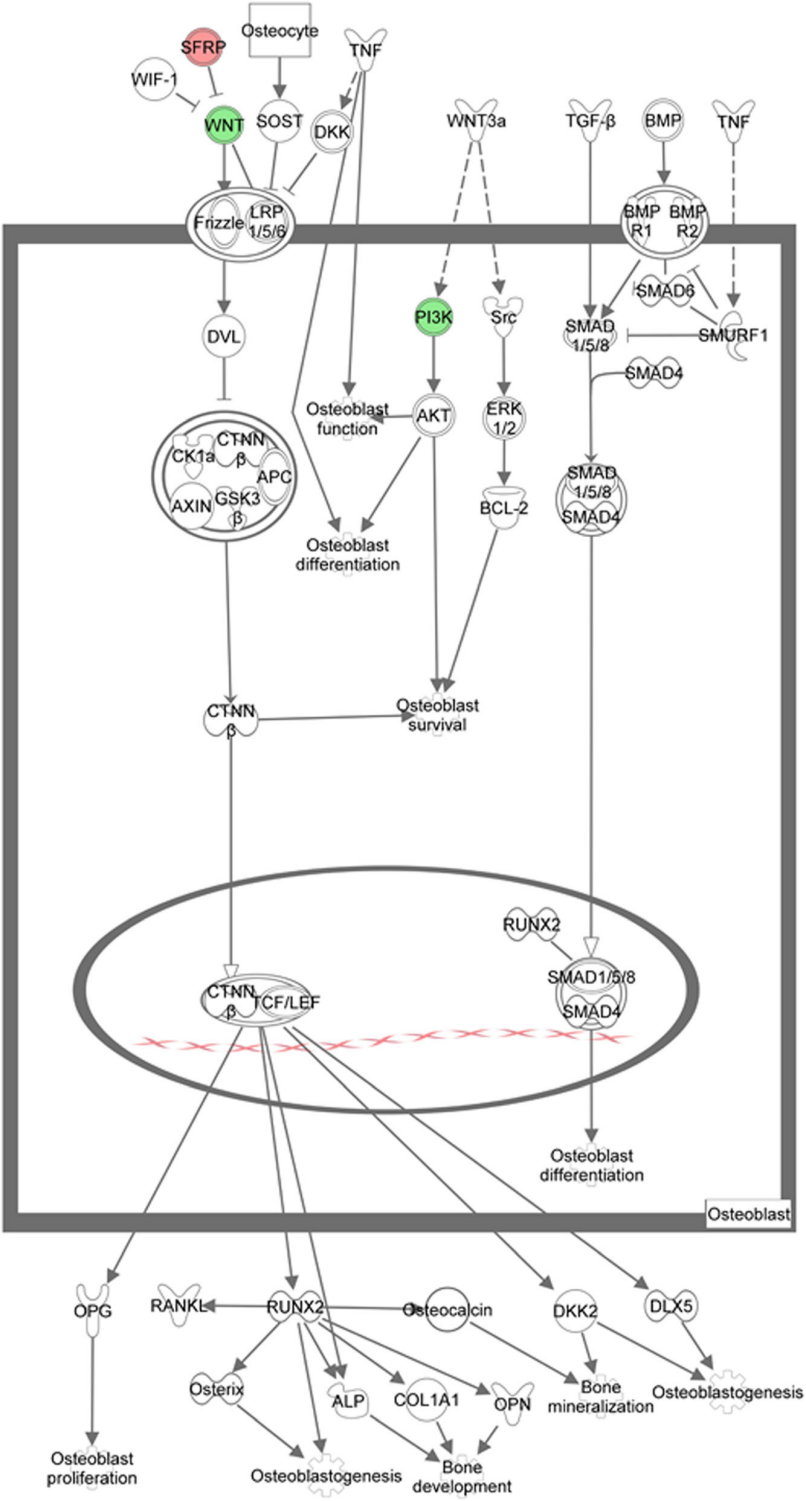
Illustration of the differential expression of mRNAs in osteoblasts using diagrams from Ingenuity Pathway Analysis (IPA). Green and red represent the up- and downregulated genes, respectively. White colored genes were the related genes introduced into the pathway

**Fig. 6:**
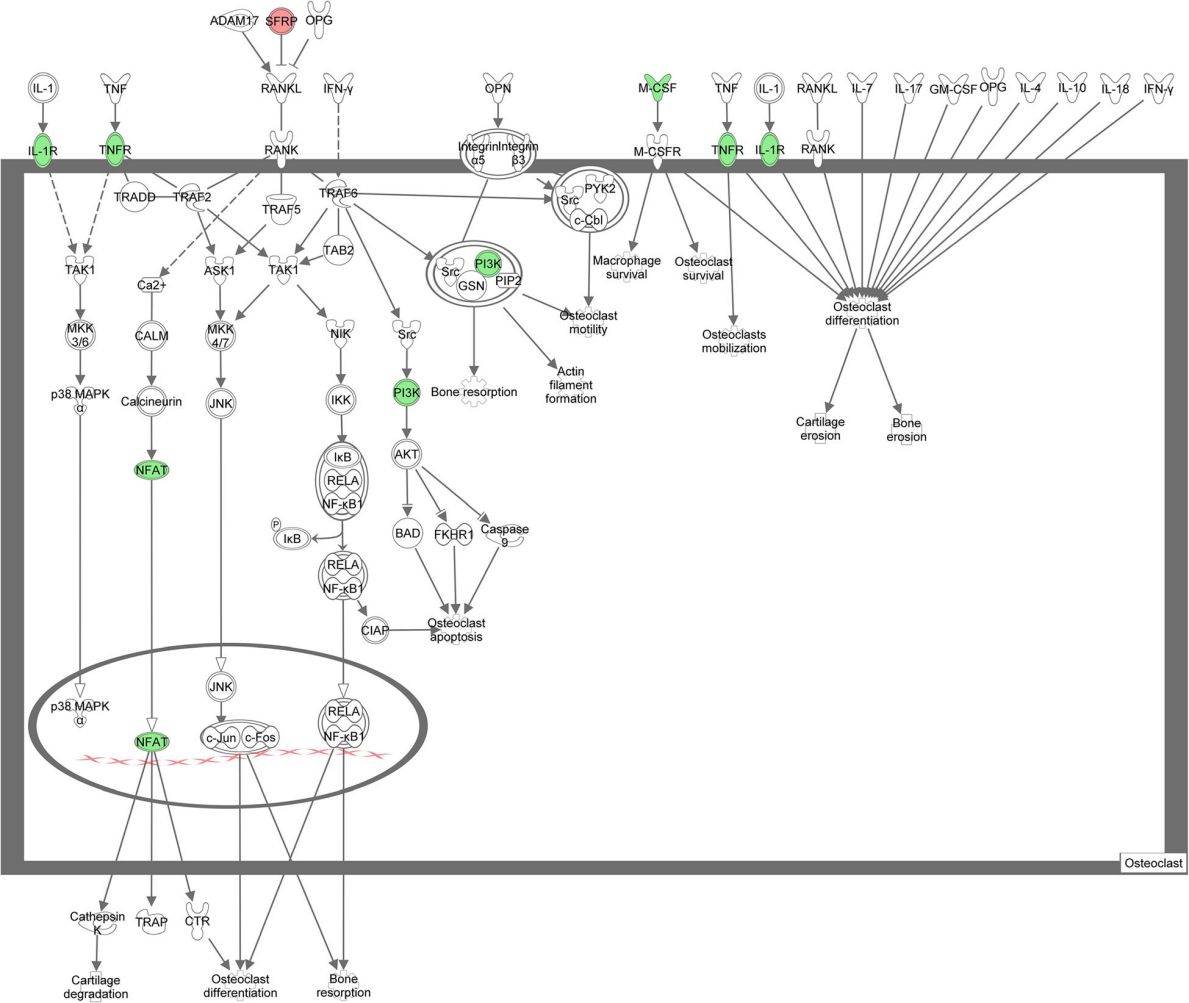
Diagram illustrating the differential expression of mRNAs in osteoclasts from Ingenuity Pathway Analysis (IPA). Green and red represent the up- and downregulated genes, respectively. White colored genes were the related genes introduced into the pathway

Eight key DE mRNAs detected from the groups of OVX and DIO were assigned to the pathway of ROOCRA in IPA (Table 2). Figures 5 and 6 illustrate the potential effects of these key mRNAs on osteoclasts and osteoblasts, respectively, on the basis of the ROOCRA pathway.

**Table 2:** Key mRNAs involved in the regulatory action of DIO on osteoclasts and osteoblasts (*p*<0.05)

Gene Symbol	Fold Change
*Sfrp1*	1.841
*Tnfrsf1a*	−1.550
*Birc3*	−1.598
*Csf1*	−1.880
*Nfatc4*	−1.936
*Il1rl1*	−2.151
*Pik3c2g*	−2.265
*Wnt9b*	−3.106

The pattern of presenting values is average value ± standard deviation (*n* = 6 for each group).

### Validation of DE circRNAs and key DE mRNAs using RT-qPCR

**Fig. 7:**
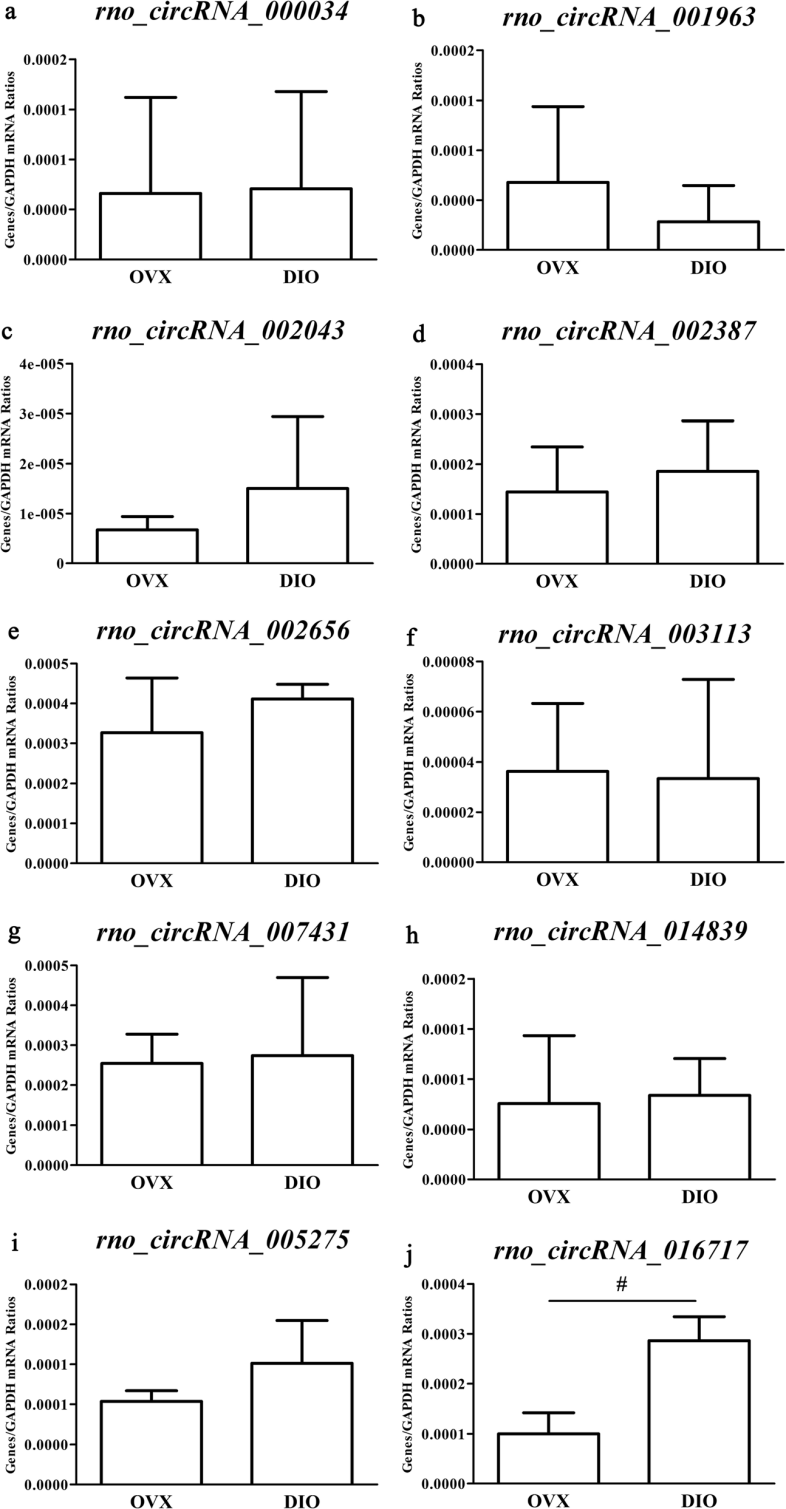
RT-qPCR validation of 10 differentially expressed circRNAs. Effect of DIO on the gene expression of rno_circRNA_000034, rno_circRNA_016717, rno_circRNA_005275, rno_circRNA_007431, rno_circRNA_003113, rno_circRNA_014839, rno_circRNA_002656, rno_circRNA_002387, rno_circRNA_002043 and rno_circRNA_001963. ^#^*p* < 0.05 vs *.*the OVX group

**Fig. 8:**
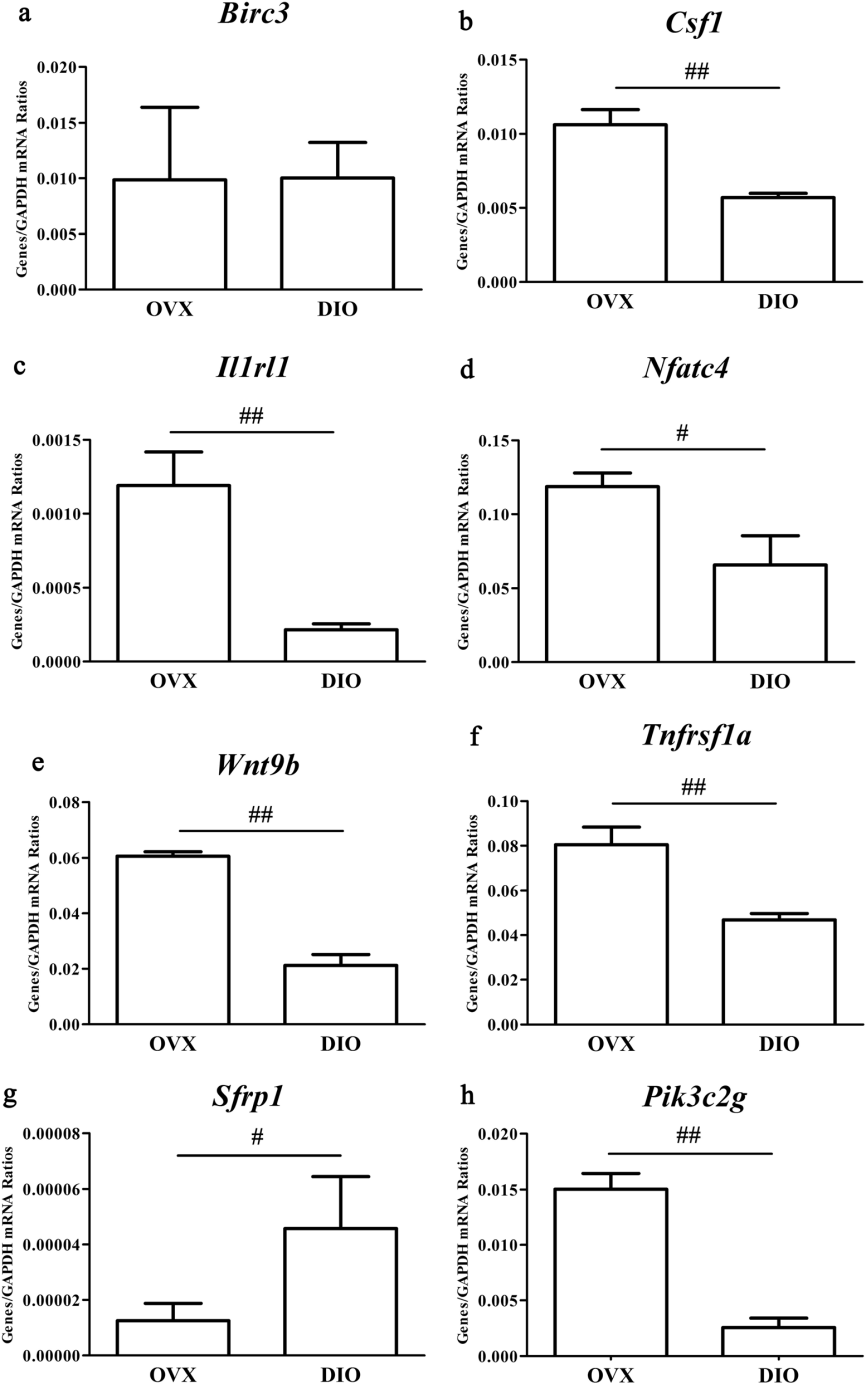
Validating 8 differentially expressed mRNAs with RT-qPCR. Roles exerted by DIO on expressions of *Birc3*, *Csf1*, *Il1rl1*, *Pik3c2g*, *Nfatc4*, *Sfrp1*, *Tnfrsf1a* and *Wnt9b*.^#^*p* < 0.05 vs. the OVX group; ^##^*p* < 0.01 vs. the OVX group

The 10 circRNA expressions are listed in Tables 1 and 8 key DE mRNA expressions listed in Table 2 were measured. As a result, rno_circRNA_016717 was the only circRNA whose RT-qPCR data were in agreement with the microarray results (Fig. 7). As for mRNAs, RT-qPCR data of all mRNAs except *Birc3* agreed with the microarray results (Fig. 8). Tables 3-4 listed the primers of DE circRNAs and key DE mRNAs for qRT-PCR experiments.

**Table 3:** Primers of differentially expressed exonic circRNAs

Gene Name	Primers
*Gapdh* (Rat)	F:5′ GCTCTCTGCTCCTCCCTGTTCTA3’
	R:5′ TGGTAACCAGGCGTCCGATA3’
rno_circRNA_002043	F:5′ CGCCCGAGAAGATTGAGAACA 3’
	R:5′ GGCTCCCACGTGCCCTTT 3’
rno_circRNA_014839	F:5′ TTCGAAGGAGGAGACTAGCAGTG 3’
	R:5′ GAACAGTTGTCAGAGGACCATCA 3’
rno_circRNA_002387	F:5′ CTATTATGTTGCACGGAGGTGG 3’
	R:5′ CCTGCTTCATACGGTGAGACA 3’
rno_circRNA_002656	F:5′ GGTTGATGAGATATTTGATGCTATA 3’
	R:5′ GAGATGAGTCCACCATTCCTTA 3’
rno_circRNA_003113	F:5′ CTTGTGGAAGAGTTTATTTCAGAGA 3’
	R:5′ GGTAGGCAGGGAAAGGTCTGT 3’
rno_circRNA_000034	F:5′ CCTCGCAAATGTGTGGTTC 3’
	R:5′ TAGCTGTTTGCACCCTGTCA 3’
rno_circRNA_007431	F:5′ ACTGCCCTGAAAAAAGGAAAGG 3’
	R:5′ TCTGGCTGAAGCTGGATTTAAAG 3’
rno_circRNA_005275	F:5′ CACCGAAACAGCAAAACAGGT 3’
	R:5′ GAAGTCCAATTTCAGTCGTCAAG 3’
rno_circRNA_001963	F:5′ AGGTTCTTTACTCAGTCCTCCAA 3’
	R:5′ CAACAACCAGCTTCCCTTGT 3’
rno_circRNA_016717	F:5′ GACTCAAAAGGATTAATAGTTAAGA 3’
	R:5′ ACTTGTTCAGGAGACGAAATG 3’

**Table 4:** Primers of key mRNAs

Gene Name	Primers
*Gapdh* (Rat)	F:5′ GCTCTCTGCTCCTCCCTGTTCTA3’
	R:5′ TGGTAACCAGGCGTCCGATA3’
*Birc3*	F:5′ GGCTACTTCAGTGGCTCCTAC 3’
	R:5′ GCCTTCTCCGTGTTCATTGC 3’
*Csf1*	F:5′ GACACCTACAGATTTTGCAGC 3’
	R:5′ CATGGTTTCCTCGATTATGACT 3’
*Il1rl1*	F:5′ GTGGACTCACCGTTACCTTCC 3’
	R:5′ GGTTAATCGCACCTCCTCTTT 3’
*Nfatc4*	F:5′ AGGAAGAGGCCGCAGTGAAC 3’
	R:5′ TCCGCCCATTGGAGACATAAA 3’
*Sfrp1*	F:5′ CTTCTACTGGCCCGAGATGC 3’
	R:5′ TGTCACACGGAGGACACACTG 3’
*Tnfrsf1a*	F:5′ CAGGTACTGCCGTGCTGTTGC 3’
	R:5′ GGCTGAAGGCTGGGATAGAGG 3’
*Wnt9b*	F:5′ GTGTGTGGTGACAACCTGAAGTA 3’
	R:5′ TGACACGCCATGACACTTGC 3’
*Pik3c2g*	F:5′ ACGGCTGCGTTCAACAAGG3’
	R:5′ TGGAAAAGCTGCCCACTCTCT3’

### A circRNA-miRNA-mRNA coexpression network showing the modulatory effect of DIO

Based on the validated DE circRNA (rno_circRNA_016717) and 7 DE key mRNAs (*Sfrp1*, *Pik3c2g*, *Wnt9b*, *Csf1*, *Il1rl1*, *Nfatc4*, and *Tnfrsf1a*), 60 potential miRNA targets were predicted using miRanda and TargetScan. Thereafter, a circRNA-miRNA-mRNA coexpression network was established that included 380 nodes and 2173 edges (Fig. S1).

**Fig. 9:**
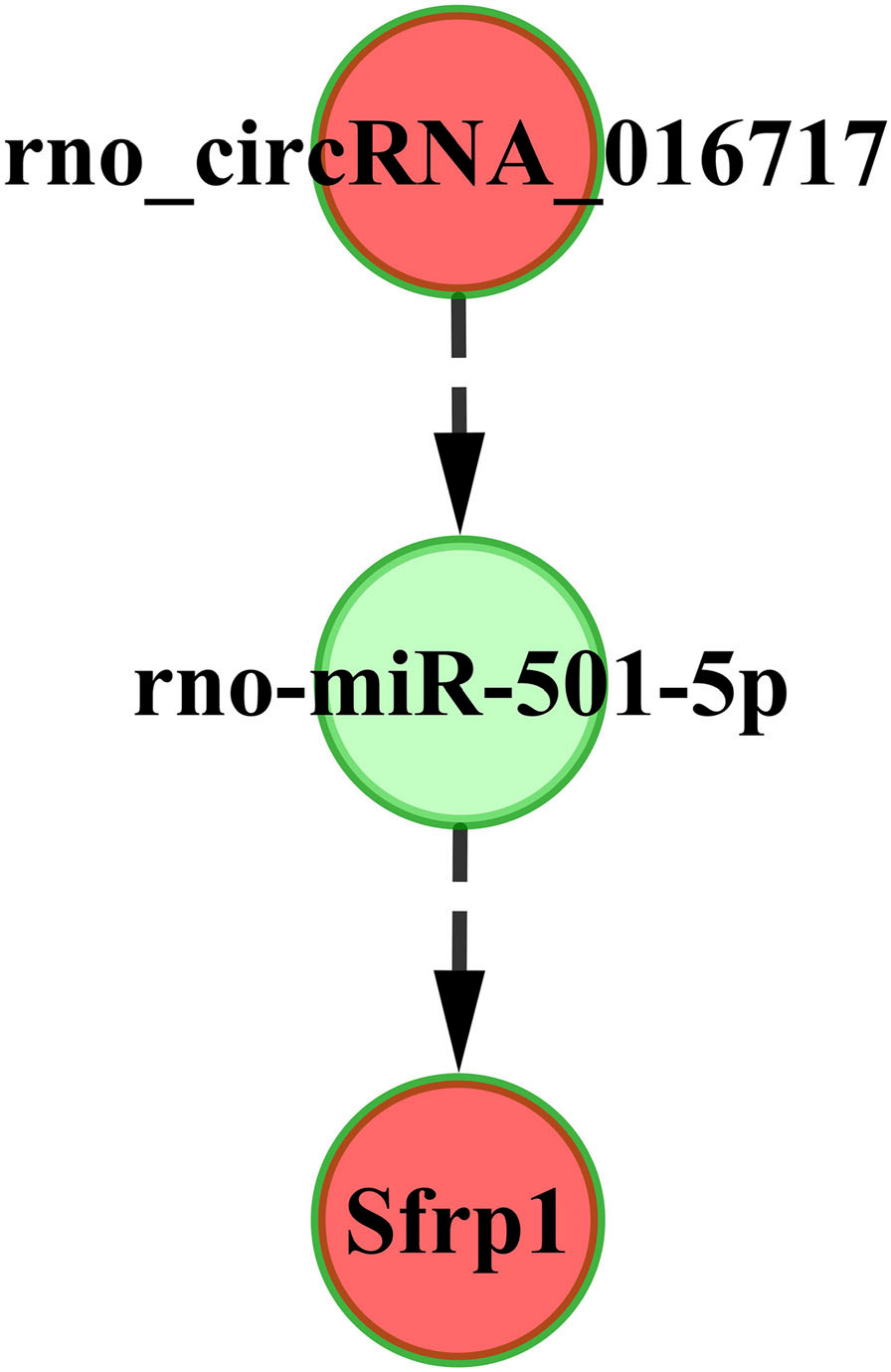
A gene axis of circRNA, miRNA and mRNA extracted from the circRNA-miRNA-mRNA network associated with the roles exerted by DIO on alveolar bone loss of rats subjected to OVX. The downregulated genes are presented in green, while the upregulated genes are presented in red

According to the interactions among circRNA, miRNAs and mRNAs, we searched for overlapping miRNAs that were downstream molecules of the DE circRNA (rno_circRNA_016717) and were upstream molecules of one DE upregulated mRNA (*Sfrp1*). Finally, only one circRNA-miRNA-mRNA axis, circRNA_016717/miR-501-5p/*Sfrp1* (Fig. 9), was explored. The roles of this axis in the regulatory effect of DIO require further intensive study.

### Validation of the predicted miRNA by RT-qPCR

**Fig. 10:**
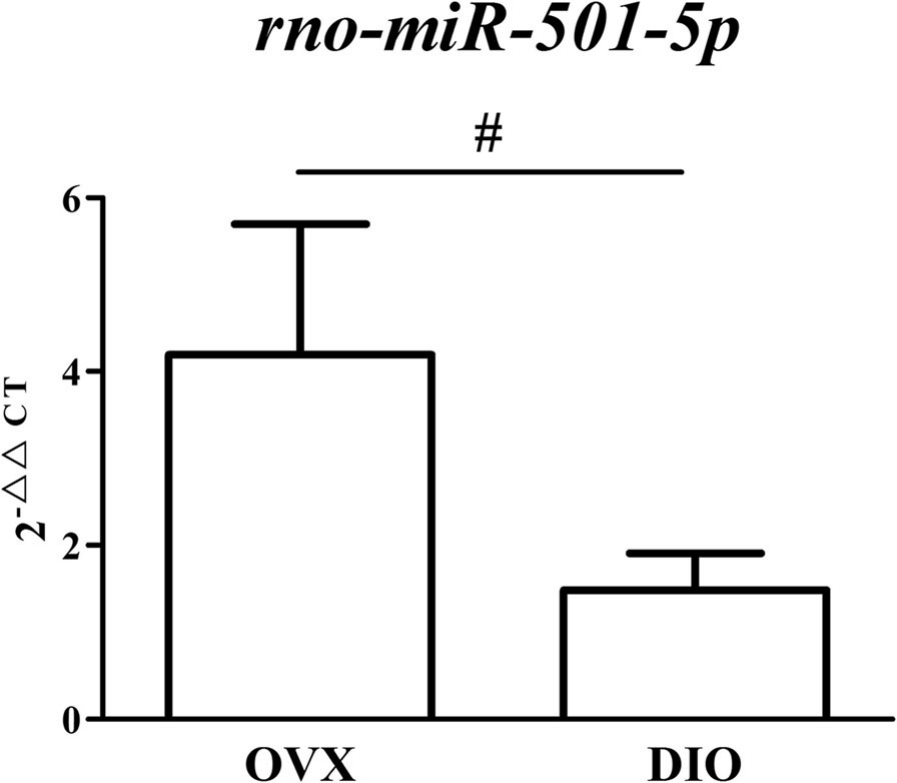
Validating the predicted miRNA with RT-qPCR. Roles exerted by DIO on expression of miR-501-5p. ^#^*p* < 0.05 vs. the OVX group

MiR-501-5p, the only predicted miRNA, was validated by RT-qPCR. As predicted, miR-501-5p expression in alveolar bone of DIO group rats was decreased compared to that in OVX rats (Fig. 10). Tables 5 listed the primers of predicted miRNA for qRT-PCR experiments.

**Table 5:** Primer of predicted miRNA

Gene Name	Primers
U6	F:5’GCTTCGGCAGCACATATACTAAAAT3’
	R:5’CGCTTCACGAATTTGCGTGTCAT3’
rno-miR-501-5p	GSP:5’GGGGGAAACCGTTACCATTAC3’
	R:5’GTGCGTGTCGTGGAGTCG3’

## Discussion

DIO is a phytoestrogen that has been shown to have an anti-osteopenic function [29, 30]; however, the mechanism of action remains unclear. Since Hansen TB et al. demonstrated that circRNAs can play a role as “miRNA sponge” [31], the important regulatory role of circRNAs on miRNAs has received increasing attention. The circRNA profile and the circRNA-miRNA-mRNA network in the protective effect of DIO on alveolar bone in OVX rats has never been reported, so we conducted a study combining microarray analysis and bioinformatics to fill the gap.

In our present study, we chose a rat model induced by OVX to mimic postmenopausal women with alveolar high-turnover bone loss. These patients usually feature high bone resorption and bone formation processes [32, 33]. Our 3-D bone microstructure modeling revealed many parameters, e.g., Tb.Th, BV/TV, BS, Tb.N, Tb.Pf, Tb.Sp, as well as SMI were significantly changed. The observation suggested that DIO and EV group rats exhibited less bone loss in alveolar bones in comparison to the model rats in the OVX group. It is worth noting that the anti-bone loss action of DIO on alveolar bone was less impactful than that of EV (Fig. 2). Our histomorphological findings in alveolar bone (Fig. 4) were largely in line with the results from micro-CT (Fig. 3).

Both TNF-alpha and OCN were upregulated in the OVX group compared to the SHAM group. However, after treatment with EV or DIO, the increased levels of the TNF-alpha and OCN were reduced (Fig. 1A and B). Our findings on EV are consistent with those in previous reports [34, 35] and strongly suggested that DIO, or other estrogens, may have a negative effect on both bone resorption and bone formation in OVX rats; thus, we confirmed that DIO can act as an estrogen-like chemical.

It is interesting to note that our histological observations showed that DIO had an anti-bone loss influence on rat alveolar bones induced by OVX, and this effect was confirmed by results from micro-CT and assay of TNF-alpha and OCN.

We conducted microarray assays to understand the role of perturbations of circRNA and mRNA profiles in the anti-bone loss action of DIO. Due to poor understanding about the function of DE circRNAs, we focused on the DE mRNAs involved in the anti-osteopenic effects of DIO. Seven validated DE mRNAs (*Sfrp1*, *Csf1*, *Il1rl1*, *Nfatc4*, *Tnfrsf1a*, *Pik3c2g* and *Wnt9b*) were associated with the ROOCRA pathway. It is clear that DIO has a multitarget inhibitory effect on the signaling pathways of both bone resorption and bone formation (Figs. 5 and 6).

The Wnt pathway is acknowledged to be one of the key signaling pathways that mediate the osteogenic differentiation not only mesenchymal stem cells but preosteoblasts [36, 37], whereas RANKL/the receptor activator of nuclear factor-kappa B (RANK) pathway plays an essential role in osteoclastogenic differentiation and bone resorption [38, 39]. Interestingly, sFRPs have dual inhibitory effects on the two pathways above. sFRPs can function as Wnt inhibitors to resemble the ligand-binding cysteine rich domain (CRD) of the Frizzled family of Wnt receptors and inhibit both canonical Wnt/beta-catenin signaling and noncanonical Wnt/planar cell polarity (PCP) signaling [40, 41]. In osteoclasts, sFRP1 can bind to RANKL directly and further block the interaction of RANKL/RANK and osteoclastogenesis [42]. DIO has been reported to inhibit breast cancer stem-like cells by deregulating the activation of Wnt/beta-catenin signaling via sFRP4 [43]. We speculated that *sFRP1 c*ould be a key target of DIO in its anti-bone loss effect.

Phosphatidylinositol 3-kinase (PI3K) controls numerous cellular functions like motility after ligand activation and cell proliferation [44]. In osteoblasts, PI3K has been well studied. PI3K inhibits osteoblast apoptosis through activating Akt (protein kinase B) and further activates PI3K/Akt by Wnt3a, and heparin promotes osteoblast differentiation [45]. For osteoclastogenesis, the PI3K/Akt pathway also plays a fundamental role. Some researchers recently reported that the activation of Akt can limit osteoclast differentiation through activating the glycogen synthase kinase 3 beta (GSK 3beta)/nuclear factor of activated T cells (NFAT) c1 signaling cascade [46]. PI3K-gamma (Pik3c2g) was also characterized recently. It has been shown that this protein is associated with the promotion of osteoclastogenesis and bone mass reduction in mice and therefore may be a potential target in osteoporosis [47]. In our present study, we inferred that DIO could attenuate osteogenesis and osteoclastogenesis by decreasing the expression of PI3K in osteoblasts and osteoclasts at the mRNA level.

Our data also showed that DIO could slow down the process of bone resorption by decreasing the signaling of three potent stimulator pathways in osteoclastogenesis (Fig. 6). DIO attenuated the ligands or receptors of macrophage colony-stimulating factor (M-CSF), interleukin-1 receptor (IL-1R) and tumor necrosis factor receptor (TNF-R) [48–50]. In particular, the results on TNF-R1 and IL-1R from the microarray assay were consistent with those of our previous study [10]. In addition, NFATC is regarded as a master transcription factor indispensable for the osteoclastogenesis induced by RANKL. The auto amplification and activation of NFATc1 give rise to a rapid upregulation of osteoclast-specific genes [51, 52] and NFATc4 was necessary for the osteoclastogenic effect of NFATc1 [53]. In this study, we surmised that TNF-R1, IL-1R and NFATc4 could be other target molecules of DIO in the reduction of bone resorption.

To sum up, the findings from the gene chip and pathway analyses suggested that the anti-bone loss action of diosgenin on alveolar bone was ascribed to inhibition of osteogenesis and osteoclastogenesis synchronously by mediating the expression of important molecules in the Wnt, PI3K, RANK/RANKL or osteoclastogenic cytokine pathways (e.g., *Sfrp1*, *Pik3c2g*, *Wnt9b*, *Csf1*, *Il1rl1*, *Nfatc4*, and *Tnfrsf1a*).

We built a circRNA-miRNA-mRNA coexpression network involved in the inhibitory action of DIO on alveolar bone loss. This network is beneficial for identifying pivotal miRNAs that are linked to the regulatory effect of rno_circRNA_016717 on the 7 key mRNAs mentioned above.

There were 380 nodes and 2173 edges in the coexpression network (Fig. S1). However, we only found one circRNA-miRNA-mRNA axis, circRNA_016717/miR-501-5p/*Sfrp1* (Fig. 9). In 2016, a study reported that miR-501-5p was considerably upregulated in human gastric cancer tissues and cell lines. The authors found that miR-501-5p may directly bind and suppress several important repressors of the Wnt/beta-catenin signaling cascade [such as GSK3 beta, dickkopf-related protein 1 (DKK1) and naked cuticle 1 (NKD1)], which lead to hyperactivated signaling in gastric cancer cells [54]. No study has reported the relationship between miR-501-5p and *Sfrp1* in vivo or in vitro, but we hypothesize that this relationship likely exists considering that *Sfrp1* is also a repressor of Wnt/beta-catenin signaling. Further efforts are needed with the aim of revealing the potential mechanism of the circRNA_016717/miR-501-5p/*Sfrp1* axis in the anti-osteoporotic effect of DIO.

## Conclusion

Our study proved that diosgenin had a protective effect on rat alveolar bone loss induced by ovariectomized. The potential mechanism of this protective effect from diosgenin was possibly associated with circRNA_016717/miR-501-5p/*Sfrp1* axis that could inhibit osteogenesis and osteoclastogenesis simultaneously by regulating the expression of important molecules in the Wnt, PI3K, RANK/RANKL or osteoclastogenic cytokine pathways.

## Supplementary information

**Additional file 1. **The differential expressions of mRNAs. In the current file, 614 mRNAs with differential expressions (*p* ≤ 0.05, fold changes ≥1.5) extracted from samples of alveolar bones from both DIO and OVX group were presented.

**Additional file 2.** A co-expression network of circRNA-miRNA-mRNA related to the roles exerted by DIO on alveolar bone loss among OVX rats. The green nodes represented key mRNAs, the blue nodes were the related mRNAs introduced into the network, the brown node represented differentially expressed circRNAs, and the red nodes represented predicted miRNAs. The lines indicate a correlation between mRNAs, circRNA and miRNAs.

## Data Availability

The current publication contains all data produced or analyzed in the study. The corresponding author will provide associated raw data which was used and/or analyzed throughout this study if requested reasonably.

## Abbreviations

ANOVA: One-way analysis of variance
BS: Bone surface
BV/TV: Trabecular bone volume fraction
circRNA: Circular RNA
CRD: Cysteine rich domain
DA: Degree of anisotropy
DE: Differentially expressed
DIO: Diosgenin
DKK1: Dickkopf-related protein 1
EDTA: Ethylenediaminetetraacetic acid
ELISA: Enzyme-linked immunosorbent assays
EV: Estradiol valerate
GSK 3beta: Glycogen synthase kinase 3 beta
IL-1R: Interleukin-1 receptor
IPKB: Ingenuity Pathways Knowledge Base
LSD: Least significant difference
M-CSF: Macrophage colony-stimulating factor
micro-CT: Microcomputed tomography
NFAT: Nuclear factor of activated T cells
NKD1: Naked cuticle 1
OPG: Osteoprotegerin
OCN: Osteocalcin
OVX: Ovariectomized
PCP: Planar cell polarity
PI3K: Phosphatidylinositol 3-kinase
RANKL: Receptor activator of nuclear factor-kappa B ligand
RANK: Receptor activator of nuclear factor-kappa B
ROOCRA: Role of Osteoblasts, Osteoclasts and Chondrocytes in Rheumatoid Arthritis
RT-qPCR: Reverse transcription-quantitative polymerase chain reaction
SHAM: Sham operation
SMI: Structural model index
Tb.Th: Trabecular thickness
Tb.Sp: Trabecular separation
Tb.Pf: Trabecular pattern factor
Tb.N: Trabecular number
TNF-alpha: Tumor necrosis factor-alpha
TNF-R: Tumor necrosis factor receptor
VOI: Volume of interest
