# Supplementary figures and images for: A circRNA-miRNA-mRNA network plays a role in the protective effect of diosgenin on alveolar bone loss in ovariectomized rats
Source: BMC Complement Med Ther. 2020 Jul 14;20:220. doi: 10.1186/s12906-020-03009-z (PMC7362493; doi:10.1186/s12906-020-03009-z)

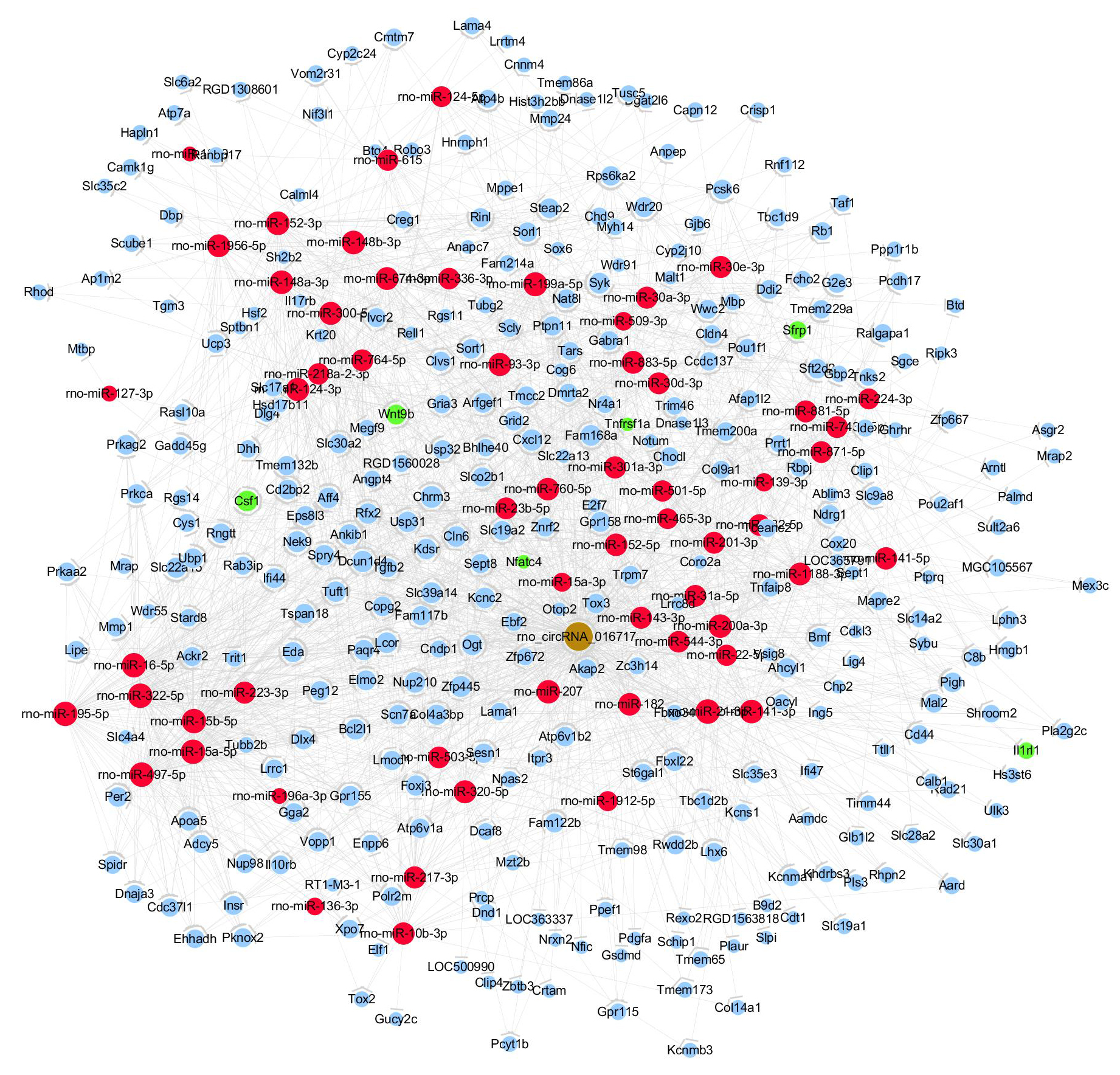

Supplement: Supplementary file 1 — Additional file 1. The differential expressions of mRNAs. In the current file, 614 mRNAs with differential expressions (p ≤ 0.05, fold changes ≥1.5) extracted from samples of alveolar bones from both DIO and OVX group were presented. [file 12906_2020_3009_MOESM1_ESM.jpg]
